# Healthy Lifestyle and Cardiac Vagal Modulation Over 10 Years: Whitehall II Cohort Study

**DOI:** 10.1161/JAHA.119.012420

**Published:** 2019-09-24

**Authors:** Vera K. Jandackova, Shaun Scholes, Annie Britton, Andrew Steptoe

**Affiliations:** ^1^ Department of Epidemiology and Public Health University of Ostrava CZ; ^2^ Department of Human Movement Studies University of Ostrava CZ; ^3^ Research Department of Epidemiology and Public Health University College London United Kingdom; ^4^ Department of Behavioural Science and Health University College London United Kingdom

**Keywords:** autonomic nervous system, heart rate variability, lifestyle, Electrophysiology, Epidemiology, Lifestyle

## Abstract

**Background:**

Increased vagal modulation is a mechanism that may partially explain the protective effect of healthy lifestyles. However, it is unclear how healthy lifestyles relate to vagal regulation longitudinally. We prospectively examined associations between a comprehensive measure of 4 important lifestyle factors and vagal modulation, indexed by heart rate variability (HRV) over 10 years.

**Methods and Results:**

The fifth (1997–1999), seventh (2002–2004), and ninth (2007–2009) phases of the UK Whitehall II cohort were analyzed. Analytical samples ranged from 2059 to 3333 (mean age: 55.7 years). A healthy lifestyle score was derived by giving participants 1 point for each healthy factor: physically active, not smoking, moderate alcohol consumption, and healthy body mass index. Two vagally mediated HRV measures were used: high‐frequency HRV and root mean square of successive differences of normal‐to‐normal R‐R intervals. Cross‐sectionally, a positively graded association was observed between the healthy lifestyle score and HRV at baseline (*P*_overall_≤0.001). Differences in HRV according to the healthy lifestyle score remained relatively stable over time. Compared with participants who hardly ever adhered to healthy lifestyles, those with consistent healthy lifestyles displayed higher high‐frequency HRV (β=0.23; 95% CI, 0.10–0.35; *P*=0.001) and higher root mean square of successive differences of normal‐to‐normal R‐R intervals (β=0.15; 95% CI, 0.07–0.22; *P*≤0.001) at follow‐up after covariate adjustment. These differences in high‐frequency HRV and root mean square of successive differences of normal‐to‐normal R‐R intervals are equivalent to ≈6 to 20 years differences in chronological age. Compared with participants who reduced their healthy lifestyle scores, those with stable scores displayed higher subsequent high‐frequency HRV (β=0.24; 95% CI, 0.01–0.48; *P*=0.046) and higher root mean square of successive differences of normal‐to‐normal R‐R intervals (β=0.15; 95% CI, 0.01–0.29; *P*=0.042).

**Conclusions:**

Maintaining healthy lifestyles is positively associated with cardiac vagal functioning, and these beneficial adaptations may be lost if not sustained.

**Clinical Perspective**

**What Is New?**

- A higher number of healthy lifestyle factors including regular physical activity, not smoking, moderate consumption of alcohol, and healthy body mass index was associated with progressively higher markers of cardiac vagal modulation in both cross‐sectional and longitudinal analyses, independently of sociodemographic factors, presence of cardiometabolic conditions, or medication use.
- A middle‐aged or older adult who hardly ever adhered to healthy behaviors over 10 years had the same level of vagally mediated heart rate variability as a person 6 to 20 years older who consistently adhered to healthy behaviors over 10 years.
- Men and women with a decreasing number of healthy lifestyle practices over the 5‐year period had lower subsequent vagally mediated heart rate variability in comparison to those with an unchanged number of healthy lifestyles.

**What Are the Clinical Implications?**

- Given that cardiac vagal modulation gradually decreases in the aging process and that dysfunction of the autonomic nervous system is a biological pathway underlying a number of cardiovascular and other age‐related diseases, sustaining a healthy lifestyle during and beyond middle age may be important in reducing age‐related deterioration of the autonomic nervous system and decreasing disease risk.

## Introduction

Having a healthy lifestyle, often characterized by a combination of regular physical activity, not smoking, moderate consumption of alcohol, and healthy body mass index (BMI), has been associated with reduced cardiovascular disease morbidity and mortality and better cardiac health.^1^, ^2^, ^3^, ^4^, ^5^ However, the pathophysiologic pathways involved are complex and not completely understood. Increased cardiac vagal modulation is thought to be one of the mechanisms that contributes to the cardioprotective effect of some aspects of healthy lifestyles.^6^, ^7^ Heart rate variability (HRV) measurement is a valid noninvasive technique for estimating autonomic nervous system (ANS) characteristics, with low HRV indicative of diminished vagal cardiovascular modulation. High‐frequency HRV (HF‐HRV) and root mean square of successive differences of normal‐to‐normal R‐R intervals (RMSSD) may be particularly important because they relate predominantly to vagal modulation of ANS. Reduced HF‐HRV and RMSSD have been identified as markers of diminished vagal cardiovascular modulations and as known predictors of cardiac morbidity and mortality.^8^, ^9^

Epidemiological studies^6^, ^7^, ^9^, ^10^ have demonstrated associations between higher vagally mediated HRV and regular physical activity, healthy BMI, not smoking, and consuming a moderate amount of alcohol, when analyzed separately, although not all studies have shown these relationships.^11^, ^12^ To our knowledge, only 1 study has comprehensively assessed the association between a healthy lifestyle (ie, the combination of behaviors) and HRV.^13^ In this cross‐sectional study of 2079 young adults,^13^ every additional healthy lifestyle factor had an incremental beneficial effect on HRV. However, cross‐sectional analyses make it difficult to estimate the long‐term associations with health behaviors and changes in lifestyle regarding cardiac vagal modulation. Moreover, assessing how cardiac vagal modulation changes longitudinally is particularly relevant in aging populations because HRV and vagal modulation gradually decrease with increasing age.^14^ Impaired ANS functioning is thought to be involved in increasing the incidence of a number of cardiovascular, mental, and age‐related diseases, including sudden cardiac death, heart failure, ventricular arrhythmias, hypertension, depression, and dementia.^15^, ^16^, ^17^, ^18^ Moreover, because the beneficial influence of healthy lifestyles mostly accumulates over a long time period, it is also inaccurate to draw conclusions from clinical trials, which often have follow‐up periods of only several months. To our knowledge, no epidemiologic study to date has prospectively examined the long‐term association between a combination of (un)healthy lifestyle factors and subsequent cardiac vagal modulation. In this study, we prospectively examined the relationship between combined healthy lifestyle factors and vagally mediated HRV over a 10‐year period using a well‐characterized population‐based cohort study. We were interested in quantifying 3 associations: (1) the association between healthy lifestyle score assessed at baseline and change in HRV over 10 years, (2) the association between habitual healthy lifestyles over 10 years and subsequent HRV, and (3) the association between changes in healthy lifestyle and subsequent HRV.

## Methods

### Study Population

Whitehall II is a cohort study of men and women initially employed by the British civil service. The target population was all London‐based office staff aged 35 to 55 years. A total of 10 308 individuals (3413 women, response rate of 73%) were initially recruited between 1985 and 1988. HRV recordings were made at the fifth (1997–1999), seventh (2002–2004), and ninth (2007–2009) data collection phases. For the purposes of the present study, phase 5 was regarded as the baseline and phase 9 was the last follow‐up occasion. The mean follow‐up time between phases 5 and 9 was 10.5 years (range: 8.9–12.3). At phase 5, all study members known to be alive and resident in the United Kingdom were invited to attend a screening clinic. Although 6554 participants (1909 women, 67%) attended the clinic, HRV was recorded for only 3365 participants because of availability of technical staff. Participants who did not undergo HRV recordings at phase 5 did not differ significantly from those who did with respect to age, sex, and employment grade.^19^

To focus on the estimation of long‐term associations, our study included individuals who participated in at least 2 of the 3 data collection phases (phases 5, 7, and 9). To maximize precision, the sample sizes for each analysis were different. The University College London ethics committee approved the study, and participants gave informed consent. Use of human material conformed to the Declaration of Helsinki. Whitehall II data, protocols, and other metadata are available to bona fide researchers for research purposes (details on the data‐sharing policy are available online^20^).

### Healthy Lifestyle

We considered 4 healthy lifestyle factors: physical activity, smoking, alcohol consumption, and BMI.

#### Physical activity

Physical activity questions consisted of 20‐items on frequency and duration of participation in walking, cycling, sports, gardening, housework, and home maintenance. Responses were combined to compute the number of hours per week spent in moderate to vigorous physical activity. Participants were categorized according to whether they adhered to the World Health Organization physical activity guidelines (at least 2.5 hours per week of moderate physical activity or >1.25 hours per week of vigorous physical activity) that are widely used and have been quantitatively validated for cardiovascular outcomes.^21^

#### Smoking status

Smoking status was categorized as current, former (>1 year), or never smoker. A healthy smoking habit was defined as not being a current smoker.

#### Alcohol consumption

Consumption of alcohol was assessed via questions on the number of alcoholic drinks (“measures” of spirits, “glasses” of wine, and “pints” of beer) consumed in the past 7 days, converted to the number of units of alcohol with each unit corresponding to 8 g of ethanol. A standard measure of spirits and a standard glass of wine were each considered to contain 8 g (1 U) of alcohol; a pint of beer was considered to contain 16 g (2 U) of alcohol. According to new UK guidelines,^22^ the healthy alcohol consumption choice was defined as being a moderate alcohol drinker (1–14 U/wk for both men and women).

#### Body mass index

BMI (kg/m^2^) was calculated from weight and height measures obtained at clinical examinations. Healthy BMI was defined as having normal BMI (18.5 to ˂25).

#### Healthy lifestyle score

To create a healthy lifestyle score for the present analyses, each healthy lifestyle factor was dichotomized as healthy versus unhealthy as follows: physically active versus somewhat active or inactive; never or former smoker versus current smoker; moderate alcohol drinking versus no or heavy drinking; and normal BMI (18.5 to ˂25) versus overweight or obese (≥25). Participants then received 1 point for every healthy criterion met, and the points were summed to obtain a healthy lifestyle score. Participants were categorized according to the healthy lifestyle score that ranged from 0 (having no healthy lifestyles) to 4 (all 4 healthy lifestyles).

### Heart Rate Variability

Details of the assessments of HRV in Whitehall II can be found elsewhere.^10^ Briefly, 5‐minute, supine, resting, 12‐lead ECGs were obtained after 5 minutes of rest. Different ECG recorders were used in the different phases. A Kardiosis device (Kardiosis Cardiologic Diagnostic Systems), a SEER MC recorder (GE Medical Systems), and a Getemed recorder (Getemed Teltow) were used at phases 5, 7, and 9, respectively. The sampling frequencies and outputs of the different devices were comparable. Using an automatic algorithm,^23^ normal sinus‐rhythm QRS complexes suited to a reliable HRV analysis were identified. Although in short‐term recordings obtained under standardized conditions, spectral HRV analysis is the preferred method,^8^ in this study both the time and frequency domains of HRV that primarily reflect vagal modulation of the ANS were analyzed: HF‐HRV (ms^2^) in the 0.15‐ to 0.4‐Hz waveband and a commonly reported time‐domain measure of HRV, RMSSD (ms). HF‐HRV was computed using a Blackman‐Tukey algorithm. Although RMSSD is highly correlated with HF‐HRV, the former may be less affected by alterations in respiration.^24^

### Covariates

Relevant confounders that have been previously shown to be associated with HRV and healthy lifestyle behaviors^10^, ^18^ included in this study were as follows: age at baseline, ethnicity (white versus other), and employment grade (high, middle, or low). Possible intermediate variables on the causal pathway between healthy lifestyles and HRV included cardiometabolic conditions and β‐blocker use.^8^, ^25^ The cardiometabolic conditions were assessed as the presence of any of the following chronic diseases or health‐related conditions: diagnosed coronary heart disease, including heart failure, stroke, and hypertension (use of hypertensive medication or blood pressure ≥140/90 mm Hg), and diabetes mellitus.

### Statistical Analyses

HRV values were logarithmically transformed to handle their skewed distributions. The Spearman rank correlation coefficient (*r*) was used to examine the correlations between heart rate and HRV measures at phases 5 and 9. We undertook the following analyses to examine 3 main healthy lifestyle and HRV associations.

#### Baseline healthy lifestyle score and change in HRV over 10 years

To examine the longitudinal association between the baseline healthy lifestyle score and change in HRV between phase 5 (1997–1999) and phase 9 (2007–2009), we used data from 2374 participants with valid information on the 4 healthy lifestyle measures and HRV at phase 5 and with at least 1 assessment of HRV data over the follow‐up period. The 2 HRV parameters (HF‐HRV and RMSSD) were analyzed in separate models. We adopted a linear mixed‐models approach by fitting the intercept as a random effect.^26^ This model included terms for the baseline healthy lifestyle score, time (exact time in years between phases, included as a continuous variable, divided by 10 to yield estimates of change over 10 years), and an interaction term between healthy lifestyle score and time to estimate the differences in the change in HRV over 10 years according to healthy lifestyle score. The cross‐sectional association is represented in the models by the main effects for the baseline healthy lifestyle score; differences in the longitudinal association by the baseline healthy lifestyle score are represented by the interaction term (healthy lifestyle score×time). Models were run sequentially. Model 1 examined the baseline healthy lifestyle score and HRV associations, adjusting for the confounding variables described above (age, sex, ethnicity, and employment grade). Model 2 additionally adjusted for the potential intermediating variables described above (cardiometabolic conditions and β‐blocker use). Within each model, the baseline healthy lifestyle score was entered as a categorical variable (3 main effects with 0 as the reference group), and the Wald test was used to test the joint hypothesis that the differences in HRV levels between the healthy lifestyle groups were simultaneously equal to zero (*P*_overall_).

#### Habitual healthy lifestyle practices over 10 years and HRV at phase 9

The association between habitual healthy lifestyles over the 10 years of follow‐up and HRV at phase 9 was estimated using an analytical sample of 3333 participants with available data on lifestyle measures at each phase (5, 7, and 9) and with HRV assessments at phase 9. To assess habitual lifestyle over the 3 assessments, we created a cumulative healthy lifestyle score that was calculated as the sum of healthy lifestyle behaviors at each study phase, ranging from 0 (no healthy lifestyles at phases 5, 7, and 9) to 12 (all 4 healthy lifestyles at phases 5, 7, and 9). According to the cumulative healthy lifestyle score, participants were categorized as hardly ever (0–4), occasionally (5–8), and always (9–12) adhering to healthy lifestyles over 10 years. We fitted general linear models to examine the association between habitual healthy lifestyle and the HRV measures at follow‐up. Model 1 examined the habitual healthy lifestyle score and HRV associations adjusting for age, sex, ethnicity, and employment grade. Model 2 additionally adjusted for cardiometabolic conditions and β‐blocker use assessed at phase 9. The joint hypothesis test for the habitual lifestyle categories (2 main effects with *hardly ever* as the reference) was conducted as described earlier.

#### Change in healthy lifestyle from phase 5 to phase 7 and subsequent HRV

The association between change in healthy lifestyles (calculated as the difference in the healthy lifestyle score between phases 5 and 7) and subsequent HRV levels was examined using an analytical sample of 2059 participants with available data on healthy lifestyles at phases 5 and 7 and with HRV assessments at phase 9. Positive values for the difference indicated a healthier lifestyle, whereas negative values indicated a less healthy lifestyle. According to the change in healthy lifestyle score, participants were categorized as those who reduced (−3 to −2), were stable unhealthy (0), were stable healthy (0), or had increased (2–4) the number of reported healthy lifestyle behaviors. The *stable unhealthy* category included those participants who reported 0 or 1 healthy lifestyle behavior at phases 5 and 7, whereas the *stable healthy* category included those with ≥2 healthy lifestyle behaviors at phases 5 and 7. We fitted general linear models to examine the association between the change in healthy lifestyles and subsequent HRV. Model 1 examined the change in healthy lifestyle score and HRV associations adjusting for age, sex, ethnicity, and employment grade and the baseline healthy lifestyle score. Model 2 additionally adjusted for cardiometabolic conditions and β‐blocker use assessed at phase 9.

We also conducted several sensitivity analyses to examine the robustness of our findings. First, to minimize the possibility of reverse causation, we restricted our analysis to a subset of “healthy” participants, namely, those who were free of coronary heart disease, heart failure, diabetes mellitus, stroke, and hypertension and had no reported β‐blocker use at any of the 3 data collection phases. Second, given the current controversy as to what constitutes healthy alcohol intake, we excluded participants who were nondrinkers at any of the 3 phases; this allowed us to dichotomize alcohol consumption as healthy (moderate drinkers: 1–14 U/wk) and unhealthy (heavy drinkers: ≥14 U/wk).

Analyses were not limited to participants with complete data on HRV and healthy lifestyle behaviors at all 3 phases. To ensure that sample differences did not account for differences in results, models were repeated using the same analytical sample for all analyses (n=1239). This had little effect on the pattern of associations, so results are presented using all available data for each analysis. Analyses were performed in Stata (v15; StataCorp).

## Results

Of the 10 308 participants at phase 1 (1985–1988) of the Whitehall II study, 306 died and 752 had dropped out from the study before the start of HRV data collection at phase 5 (1997–1999). Of the 9250 remaining participants, 2374 participants had valid information on the 4 healthy lifestyle measures and HRV at phase 5 and had also participated in at least 1 other wave over follow‐up. Descriptive characteristics of this sample (n=2374) are presented in Table 1. The average (mean±SD) age was 55.4±6 years, and 72% of participants were male. Most participants had 2 (41%) or 3 (27%) healthy lifestyles. The number of participants with a presence of any cardiometabolic condition including coronary heart disease, stroke, heart failure, diabetes mellitus, and hypertension increased from 925 (38.9%) to 1234 (52.2%) from phases 5 to 9. Strong positive Spearman rank correlations were found between HF‐HRV and RMSSD at both phases 5 and 9 (*r*=0.93 and *r*=0.94, respectively). Correlations between HRV measures and heart rate were negative and lower in magnitude at phases 5 and 9, respectively (HF‐HRV: *r*=−0.44 and *r*=−0.35; RMSSD: *r*=−0.57 and *r*=−0.44).

**Table 1:** Descriptive Characteristics of the Sample^a^ at Baseline (Phase 5; 1997–1999; n=2374)

Characteristic	Result
Age, y, mean (SD)	55.4 (5.9)
Men	1719 (72.3)
White ethnic origin	2201 (92.6)
Low employment grade	289 (12.2)
Heart rate, beats/min (SE)	69.1 (0.2)
HF‐HRV, ms^2^ (SE)^b^	124.8 (1.0)
RMSSD, ms (SE)^b^	20.2 (1.0)
Not a current smoker	2184 (91.8)
Meeting physical activity guidelines	608 (25.6)
Moderate alcohol intake (1–14 U/wk)	1219 (51.4)
Healthy BMI (18.5–25)	975 (41)
No. of healthy lifestyles	
0	55 (2.3)
1	576 (24.3)
2	963 (40.6)
3	640 (26.9)
4	140 (5.9)
Healthy lifestyle score, mean (SD)	2.2 (0.9)
Cardiometabolic condition^c^	925 (38.9)
Β‐blocker use	106 (4.5)

Data shown as n (%) except as noted. BMI indicates body mass index; HF‐HRV, high‐frequency heart rate variability; RMSSD, root mean square of successive differences of normal‐to‐normal R‐R intervals.

a In those who have data on HRV and healthy lifestyles at phase 5 and who participated in at least 1 other wave over follow‐up.

b Age‐adjusted geometric mean and standard error.

c Presence of any of the following cardiometabolic conditions: diagnosed coronary heart disease, stroke, heart failure, diabetes mellitus, and hypertension (use of hypertensive medication or blood pressure ≥140/90 mm Hg).

### Baseline Healthy Lifestyle Score and Change in HRV Over 10 Years

Cross‐sectionally, a significant graded association was observed between a higher baseline healthy lifestyle score and baseline HF‐HRV and RMSSD (*P*_overall_≤0.001). Compared with participants with a healthy lifestyle score of 0, those with 3 or 4 healthy lifestyle behaviors at baseline had significantly higher HF‐HRV (3 behaviors: β=0.48; 95% CI, 0.17–0.79; *P*=0.003; 4 behaviors: β=0.56; 95% CI, 0.21–0.92; *P*=0.002) and higher RMSSD (3 behaviors: β=0.25; 95% CI, 0.07–0.43; *P*=0.005; 4 behaviors: β=0.30; 95% CI, 0.10–0.50; *P*=0.003) after adjustment for age, sex, ethnicity, employment grade, the presence of cardiometabolic conditions, and β‐blocker use (Table 2; model 2). In the analysis using the baseline healthy lifestyle score as a single continuous variable, the fully adjusted β coefficients representing the change in HRV associated with a 1‐point increase in the healthy lifestyle score were 0.15 (95% CI, 0.10–0.20; *P*≤0.001) for HF‐HRV and 0.08 (95% CI, 0.06–0.11; *P*≤0.001) for RMSSD (data not shown).

**Table 2:** Associations (Mixed‐Models Analyses) Between Healthy Lifestyle Score at Baseline and Log‐Transformed HRV Measures Over 10 Years (n=2374)

	Log HF‐HRV		Log RMSSD	
	Model 1	Model 2	Model 1	Model 2
β (95% CI) for cross‐sectional associations				
Healthy lifestyle score				
0	Reference	Reference	Reference	Reference
1	0.17 (−0.14 to 0.49)	0.17 (−0.14 to 0.49)	0.08 (−0.10 to 0.26)	0.08 (−0.10 to 0.25)
2	0.25 (−0.06 to 0.56)	0.25 (−0.06 to 0.56)	0.13 (−0.05 to 0.30)	0.12 (−0.05 to 0.30)
3	0.49 (0.17–0.80)^a^	0.48 (0.17–0.79)^a^	0.26 (0.08–0.44)^a^	0.25 (0.07–0.43)^a^
4	0.57 (0.22–0.93)^a^	0.56 (0.21–0.92)^a^	0.31 (0.11–0.51)^a^	0.30 (0.10–0.50)^a^
*P*_overall_	≤0.001	≤0.001	≤0.001	≤0.001
β (95% CI) for longitudinal associations				
Healthy lifestyle score×time				
0	Reference	Reference	Reference	Reference
1	−0.27 (−0.74 to 0.20)	−0.26 (−0.73 to 0.20)	−0.09 (−0.36 to 0.18)	−0.08 (−0.35 to 0.19)
2	−0.29 (−0.75 to 0.17)	−0.28 (−0.74 to 0.17)	−0.10 (−0.37 to 0.17)	−0.09 (−0.35 to 0.18)
3	−0.43 (−0.90 to 0.03)	−0.42 (−0.89 to 0.04)	−0.20 (−0.47 to 0.07)	−0.18 (−0.45 to 0.09)
4	−0.58 (−1.10 to −0.05)^a^	−0.58 (−1.10 to −0.06)^a^	−0.24 (−0.55 to 0.06)	−0.23 (−0.54 to 0.07)
*P*_overall_	0.07	0.06	0.09	0.10

The cross‐sectional association is represented by main effects for baseline healthy lifestyle (0 as reference) adjusting for time, age, and other covariates. The longitudinal association is represented by the terms for the interaction between baseline healthy lifestyle and time. Model 1: adjusted for age, sex, ethnicity, and employment grade. Model 2: adjusted for age, sex, ethnicity, employment grade, presence of any cardiometabolic condition, and β‐blocker use. HF‐HRV indicates high‐frequency heart rate variability; HRV, heart rate variability; RMSSD, root mean square of successive differences of normal‐to‐normal R‐R intervals.

a *P*≤0.05.

Longitudinally, there was an estimated decrease in both HRV markers from baseline to follow‐up: log HF‐HRV decreased from 4.82 to 4.49 (*P*≤0.001) and log RMSSD decreased from 3.01 to 2.94 (*P*≤0.001), corresponding to a decrease on the original scale of 34.6 ms^2^ (28%) in HF‐HRV and a decrease of 1.37 ms (7%) in RMSSD over the 10 year follow‐up period (estimated from the fully adjusted model). Marginal evidence from the longitudinal associations (healthy lifestyle score×time) suggested that the differences in log HF‐HRV (*P*_overall_=0.06) or log RMSSD (*P*_overall_ *=*0.10) according to the grouped baseline healthy lifestyle score became smaller over follow‐up (Table 2; model 2).

### Habitual Lifestyle Over 10 Years and HRV at Phase 9

There were significant differences in HF‐HRV and RMSSD at follow‐up, according to the habitual healthy lifestyle score over 10 years (*P*_overall_=0.001 and *P*_overall_≤0.001, respectively; Table 3 and Figure). Compared with participants who hardly ever adhered to a healthy lifestyle over 10‐year follow‐up, participants who always followed healthy lifestyle practices displayed higher HF‐HRV (β=0.23; 95% CI, 0.10–0.35; *P*=0.001) and higher RMSSD (β=0.15;95% CI, 0.07–0.22; *P*=0.001) at follow‐up after adjustment for age, sex, ethnicity, employment grade, the presence of cardiometabolic conditions, and β‐blocker use (model 2 in Table 3 and Figure). These coefficients correspond to a fully adjusted difference on the original scale of 21.7 ms^2^ in HF‐HRV and of 2.8 ms in RMSSD between participants who adhered to healthy lifestyles throughout the time period compared with those who hardly ever adhered to healthy lifestyles over the 10 years. Because the average age‐adjusted decreases in HF‐HRV and RMSSD were 34.6 ms^2^ and 1.37 ms, respectively, over 10 years, the differences in HRV are equivalent to a different of ≈6 to 20 years in chronological age between individuals who hardly ever adhered to a healthy lifestyle and those who always followed healthy lifestyle practices.

**Table 3:** Habitual Healthy Lifestyle Over 10 Years and HRV Measures at Follow‐Up (n=3333)

Habitual Healthy Lifestyle Over 10 Years^a^	Log HF‐HRV		Log RMSSD	
	Model 1, β (95% CI)	Model 2, β (95% CI)	Model 1,β (95% CI)	Model 2,β (95% CI)
Hardly ever (n=570)	Reference	Reference	Reference	Reference
Occasionally (n=1963)	0.05 (−0.05 to 0.16)	0.05 (−0.06 to 0.15)	0.04 (−0.02 to 0.10)	0.04 (−0.02 to 0.10)
Always (n=800)	0.24 (0.11–0.37)^b^	0.23 (0.10–0.35)^b^	0.15 (0.07–0.22)^b^	0.15 (0.07–0.22)^b^
*P*_overall_	≤0.001	0.001	≤0.001	≤0.001

Model 1: adjusted for age, sex, ethnicity, and employment grade. Model 2: adjusted for age, sex, ethnicity, employment grade, presence of any cardiometabolic condition, and β‐blockers use. HF‐HRV indicates high‐frequency heart rate variability; HRV, heart rate variability; RMSSD, root mean square of successive differences of normal‐to‐normal R‐R intervals.

a Habitual lifestyle over 10 years; *hardly ever*,* occasionally*, and *always* include those participants who reported 0–4, 5–8, and 9–12 healthy lifestyle behaviors (not currently smoking, physically active, moderate alcohol consumption, and healthy body mass index), respectively, at the 3 time points (phases 5, 7, and 9).

b *P*≤0.001.

**Figure 1:**
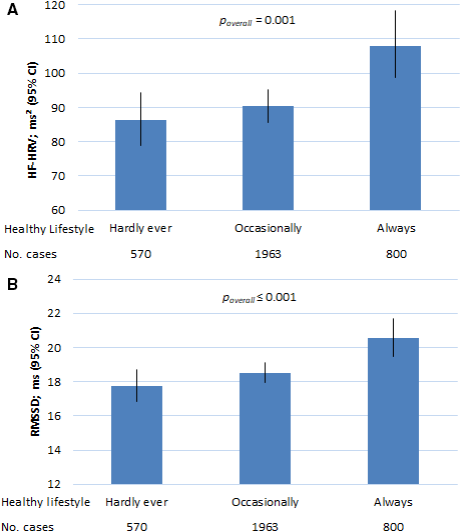
Predicted HF‐HRV (**A**) and RMSSD (**B**) at follow‐up according to habitual healthy lifestyle over 10 years (n=3333). The HF‐HRV and RMSSD values for each category of habitual healthy lifestyle were predictions from a general linear model including category of habitual lifestyle based on healthy lifestyle score over 10 years, age at baseline, sex, employment grade, presence of any cardiometabolic condition, and use of β‐blockers. Predicted values for HF‐HRV and RMSSD are the geometric means and SEs; general linear model. *P* values are based on the Wald test of the joint hypothesis. Habitual healthy lifestyle practices over 10 years are defined as *hardly ever, occasionally*, and *always* including those participants who reported 0–4, 5–8, and 9–12 healthy lifestyle factors (not currently smoking, physically active, moderate alcohol consumption, and healthy body mass index), respectively, out of the time points (phases 5, 7, and 9). HF‐HRV indicates high‐frequency heart rate variability; RMSSD, root mean square of successive differences of normal‐to‐normal R‐R intervals.

### Change in Healthy Lifestyles Between Phases 5 and 7 and Subsequent HRV

Compared with participants who reduced their healthy lifestyle score by at least 2 points from phase 5 to phase 7, those participants who had a stable healthy lifestyle score displayed higher subsequent HF‐HRV (β=0.24; 95% CI, 0.01–0.48; *P*=0.046) and higher subsequent RMSSD (β=0.15; 95% CI, 0.01–0.29; *P*=0.042) after adjustment for age, sex, ethnicity, employment grade, healthy lifestyle score at phase 5, presence of any cardiometabolic condition, and β‐blocker use at phase 9 (Table 4; model 2). Compared with participants who reduced their healthy lifestyle score by at least 2 points from phase 5 to phase 7, those who increased their healthy lifestyle score by at least 2 points over the same time period had higher subsequent RMSSD (β=0.24; 95% CI, 0.02–0.46; *P*=0.033) after adjustment for all covariates (Table 4; model 2).

**Table 4:** Change in Healthy Lifestyle From Phase 5 to 7 and HRV Measures at Phase 9 (n=2059)

Change^a^ in Healthy Lifestyle Score	Log HF‐HRV		Log RMSSD	
	Model 1, β (95% CI)	Model 2, β (95% CI)	Model 1, β (95% CI)	Model 2, β (95% CI)
Deterioration (n=123)	Reference	Reference	Reference	Reference
Stable unhealthy (n=484)	0.24 (−0.09 to 0.58)	0.23 (−0.10 to 0.56)	0.13 (−0.07 to 0.32)	0.13 (−0.06 to 0.33)
Stable healthy (n=1349)	0.25 (0.01–0.49)^b^	0.24 (0.01–0.48)^b^	0.14 (−0.01 to 0.28)	0.15 (0.01–0.29)^b^
Improvement (n=103)	0.35 (−0.02 to 0.73)	0.36 (−0.02 to 0.74)	0.22 (−0.01 to 0.44)	0.24 (0.02–0.46)^b^
*P*_overall_	0.17	0.16	0.17	0.09

Model 1: adjusted for age, sex, ethnicity, employment grade, and healthy lifestyle score at phase 5. Model 2: adjusted for age, sex, ethnicity, employment grade, healthy lifestyle score at phase 5, presence of any cardiometabolic condition, and β‐blockers use at phase 9. HF‐HRV indicates high‐frequency heart rate variability; HRV, heart rate variability; RMSSD, root mean square of successive differences of normal‐to‐normal R‐R intervals.

a A deterioration and improvement represents a change of at least 2 points in the healthy lifestyle score over 5 years. The *stable unhealthy* category included those participants who reported no or 1 healthy lifestyle behavior at phases 5 and 7. The *stable healthy* category included those participants who reported ≥2 healthy lifestyle behaviors at phases 5 and 7.

b *P*≤0.05.

In Tables S1 through S3, we provide results on the associations between healthy lifestyles and heart rate from our 3 main analyses.

### Sensitivity Analyses

The results of our primary analyses were not appreciably altered in the sensitivity analyses which excluded participants with the presence of any cardiometabolic conditions at phase 5, 7, or 9 (ie, restricting our analysis to a subset of healthy participants) and those not consuming any alcohol at any of the 3 data collection phases. Next we present the *P* values from the sensitivity analyses based on the fully adjusted models.

First, for the analysis of baseline healthy lifestyle and change in HRV, the positive graded association between higher number of healthy lifestyles and baseline HRV measures remained strongly significant in the subgroup of healthy individuals (*P*_overall_=0.003). In addition, the differences in HRV according to the grouped baseline healthy lifestyle score persisted over time both in the healthy subgroup (*P*_overall_=0.45; n=1094) and in the subgroup of moderate or heavy alcohol consumers (*P*_overall_=0.06; n=1740).

Second, for the analysis of habitual lifestyles and subsequent HRV, habitual healthy lifestyle over 10 years was associated with higher HRV measures at follow‐up in the subgroup of 1588 healthy individuals (*P*_overall_≤0.010) and in the subgroup of 2491 moderate or heavy alcohol consumers (*P*_overall_≤0.004).

## Discussion

Our investigation based on a large population sample of middle‐aged and older adults suggests that higher numbers of healthy lifestyle practices were associated with more favorable vagally mediated HRV in both cross‐sectional and longitudinal associations. Our study produced a number of main findings. First, an increasingly healthy lifestyle at baseline was related to progressively increasing HRV, and this difference remained relatively stable over time. Second, participants consistently adhering to healthy lifestyle practices over the 10‐year period had higher HRV at follow‐up compared with those who hardly ever adhered to a healthy lifestyle over 10 years. Third, participants with a decreased number of healthy lifestyle practices over the 5‐year period (phase 5 to phase 7) had lower subsequent HRV in comparison with those with an unchanged number of healthy lifestyles. All of these associations were independent of sociodemographic factors, the presence of cardiometabolic conditions, and/or β‐blocker use. These associations were present in a subset of healthy participants and after excluding nondrinkers. To our knowledge, this large, prospective, population‐based study is the first to demonstrate an association between a combination of healthy lifestyle behaviors with indexes of vagal modulation over a long period of time in middle‐aged and older adults.

The strong and graded association between the number of healthy lifestyle practices and HRV measures in our cross‐sectional analysis suggests that having any healthy lifestyle practice is better than having none, and having more is better. A similar finding was observed in the cross‐sectional study described by Aeschbacher et al^13^ using 24‐hour HRV recording and healthy lifestyle indicators. Given that certain healthy lifestyle factors show stronger associations with decreased autonomic functioning and risk of disease than others, it could be argued that simply adding the lifestyle factors to obtain a combined summary score may lead to misclassification with heterogeneity among participants having the same healthy lifestyle score. However, previous studies^2^, ^27^ have demonstrated that the relative weighting of lifestyle factors did not have an impact on the associations between healthy lifestyles and health outcomes, suggesting that the adoption of an overall healthy lifestyle in which these healthy lifestyle habits are aggregated is optimal.

The number of healthy lifestyle practices was not related to different rates of HRV decline over 10 years. This result is in agreement with our recent findings that HRV decreases naturally as a result of normal aging processes.^14^ However, individuals with healthier lifestyles always demonstrated higher HRV at follow‐up than those with poorer lifestyles. The evidence that unhealthy lifestyle factors such as smoking, physical inactivity, nonmoderate amounts of alcohol consumption, and being overweight or obese negatively affect health is overwhelming. However, these factors have been examined in combination only in the past decade. Large epidemiological studies have shown that the combined impact of healthy lifestyle behaviors on various health outcomes, including heart failure,^2^ coronary heart disease,^27^ diabetes mellitus, cancer, and all cause mortality,^28^, ^29^ is much larger than the effect of separate lifestyle factors. For example, a meta‐analysis of prospective studies^5^ showed that a combination of at least 4 healthy lifestyle factors (versus no healthy lifestyles) is associated with a reduction of all‐cause mortality risk by 66%, whereas having just 1 healthy behavior (versus 0) reduced the all‐cause mortality risk by only 28%. In a large prospective study,^4^ 4 health behaviors in combination predicted a 4‐fold difference in total mortality in men and women, with an estimated impact equivalent to a 14‐year difference in chronological age. In our study, the estimated difference in HF‐HRV and RMSSD between those adhering consistently and those who hardly ever adhered to healthy behaviors over 10 years corresponded to a 6‐ to 20‐year difference in chronological age.

Our finding that changes in lifestyle over 5 years were associated with subsequent HRV complements results from clinical and experimental studies.^30^, ^31^ For example, in patients successfully rehabilitated after a first myocardial infarction, an increase in vagally mediated HRV (HF‐HRV) was observed in those who adhered to the prescriptions involving a regular exercise program, healthy diet, and smoking avoidance in the long term. In patients who failed to maintain healthy lifestyle practices, the increase in parasympathetic tone induced during rehabilitation was lost after 1 year.^30^

The exact mechanisms that connect healthy lifestyle with ANS activity and health are complex and are not completely understood. One explanation for the beneficial effect of regular physical activity on ANS function may be positive functional adaptations of central regulatory control and improvement in baroreflex, respiratory, and circadian fluctuations.^7^, ^32^ Regular physical activity may also directly affect cardiomyocytes, improving contractile capacity and cardiac electric stability.^33^, ^34^ Regular cigarette consumption leads to decreased vagal modulation and increased sympathetic nerve activity that becomes persistent via a positive feedback loop between sympathetic activity and reactive oxidative stress. Furthermore, baroreflex suppression of sympathetic activation seems to be attenuated in habitual smokers, allowing sympathetic excitation to occur without restraint in the setting of increased pressor responses.^35^ Among overweight and obese persons, and in persons with unhealthy diets, HRV tends to be lower than among persons with healthy weight and healthy diets.^36^ Some evidence also suggests that high levels of body fat, especially visceral fat, are independently associated with reduced HRV.^37^ The biological mechanism for decreased HRV with chronic, heavy alcohol use may result from impaired central inhibitory feedback control of the parasympathetic branch of the ANS^38^ or from toxic damage to the vagus nerve.^39^ Loss of protective vagal reflexes seems to impair physical and psychological functioning and capacity to respond flexibly to efferent stimuli, resulting in increased disease vulnerability.^15^ Consequently, the increased modulation of parasympathetic activity associated with the combination of healthy behaviors might contribute to the beneficial effects on visceral organs and on the brain. Improving health through better vagal modulation implemented through the adoption and maintenance of a healthy lifestyle should be targets for preventive and intervention strategies.

## Strengths and Limitations

A key strength of our investigation is the use of participant‐level longitudinal data with 3 repeated measures of HRV over a decade in a large nonclinical population. The Whitehall II study is an occupation‐based cohort and thus is healthier on average than the general population. Nevertheless, etiological findings from the Whitehall II cohort have been shown to be comparable to other population‐based studies.^40^

## Conclusions

Maintaining a healthy lifestyle through the transition from middle or older ages associates positively with cardiac vagal functioning at a later stage of life, and these beneficial adaptations may be lost if healthy practices are not sustained. Given that cardiac vagal modulation gradually decreases in the aging process and that the dysfunction of the ANS is a biological pathway related to a number of diseases, having and sustaining a healthy lifestyle may play an important preventive role in reducing age‐related disease risk.

## Sources of Funding

This work was supported by the Czech Science Foundation (registration number: GACR17‐22346Y to Jandackova), by a University of Ostrava Award (SGS18/LF/2016‐2017 to Jandackova), and by the British Heart Foundation (to Steptoe). The Whitehall II study is supported by grants from the Medical Research Council; British Heart Foundation; Health and Safety Executive; Department of Health; National Heart, Lung and Blood Institute (HL36310; US National Institutes of Health [NIH]); National Institute on Aging (NIH); Agency for Health Care Policy Research (HS06516); and the JD and CT MacArthur Foundation Research Networks on Successful Midlife Development and Socioeconomic Status and Health.

## Disclosures

None.

## Supporting information


**Table S1.** Associations (Mixed‐Models Analyses) Between Healthy Lifestyle Score at Baseline and Heart Rate Over 10 Years (n=2374)
**Table S2.** Habitual Healthy Lifestyle Over 10 Years and Heart Rate at Follow‐Up (n=3333)
**Table S3**. Change in Lifestyle From Phase 5 to 7 and Heart Rate at Phase 9 (n=2059)
