# Supplementary material for: Healthy Lifestyle and Cardiac Vagal Modulation Over 10 Years: Whitehall II Cohort Study
Source: J Am Heart Assoc. 2019 Sep 24;8(19):e012420. doi: 10.1161/JAHA.119.012420 (PMC6806037; doi:10.1161/JAHA.119.012420)
Supplement: Supplementary file 1 — Table S1. Associations (Mixed‐Models Analyses) Between Healthy Lifestyle Score at Baseline and Heart Rate Over 10 Years (n=2374) Table S2. Habitual Healthy Lifestyle Over 10 Years and Heart Rate at Follow‐Up (n=3333) Table S3. Change in Lifestyle From Phase 5 to 7 and Heart Rate at Phase 9 (n=2059) [file JAH3-8-e012420-s001.pdf]

# SUPPLEMENTAL MATERIAL

**Table S1. Associations (Mixed-Models Analyses) Between Healthy Lifestyle Score at Baseline and Heart Rate over 10 years (n=2, 374).**

| | **Heart rate** | | | |
|---|---|---|---|---|
| | **β (95% CI) for Cross-sectional Associations** | | **β (95% CI) for Longitudinal Associations** | |
| | **Model 1** | **Model 2** | **Model 1** | **Model 2** |
| **Healthy Lifestyle Score** | | | | |
| **0** | reference | reference | reference | reference |
| **1** | -1.01 (-3.96 to 1.94) | -0.72 (-3.57 to 2.14) | 1.13 (-2.33 to 4.60) | 0.52 (-2.70 to 3.74) |
| **2** | -1.55 (-4.45 to 1.36) | -1.30 (-4.11 to 1.50) | 1.57 (-1.84 to 4.97) | 0.76 (-2.41 to 3.92) |
| **3** | -4.47 (-7.41 to -1.53)* | -4.29 (-7.13 to -1.45)* | 2.49 (-0.96 to 5.95) | 1.49 (-1.72 to 4.70) |
| **4** | -6.84 (-10.17 to -3.50)** | -6.65 (-9.88 to -3.42)** | 3.04 (-0.84 to 6.92) | 2.32 (-1.29 to 5.92) |
| $p_{overall}$ | ≤ 0.001 | ≤ 0.001 | 0.169 | 0.313 |

The cross-sectional association is represented by main effects for healthy lifestyle adjusting for time, age and other covariates. The longitudinal association is represented by the term for the interaction between healthy lifestyle and time.

Model 1: adjusted for age, sex, ethnicity and employment grade

Model 2: adjusted for age, sex, ethnicity, employment grade, presence of any cardiometabolic condition and betablocker use

$^{**}$ $p \leq 0.001$; $^{*}$ $p \leq 0.05$

**Table S2. Habitual Healthy Lifestyle over 10 years and Heart Rate at follow-up (n=3333).**

| | **Heart rate** | |
|---|---|---|
| **Habitual Healthy Lifestyle over 10 years**† | **Model 1**<br>**β (95% CI)** | **Model 2**<br>**β (95% CI)** |
| Hardly ever (n=570) | reference | reference |
| Occasionally (n=1,963) | -1.77 (-2.71 to -0.83)** | -1.90 (-2.81 to -0.10)** |
| Always (n=800) | -4.61 (-5.76 to -3.45)** | -4.97 (-6.09 to -3.85)** |
| $p_{overall}$ | ≤0.001 | ≤0.001 |

Model 1: adjusted for age, sex, ethnicity and employment grade

Model 2: adjusted for age, sex, ethnicity, employment grade, presence of any cardiometabolic condition and betablockers use

†Habitual Lifestyle over 10 years; „hardly ever", „occasionally" and „always" includes those participants who reported 0-4, 5-8 and 9-12 healthy lifestyle behavior (not currently smoking, physically active, moderate alcohol consumption and healthy BMI) respectively at the three time points (phases 5, 7 and 9)

** $p \leq 0.001$

**Table S3. Change in Lifestyle from Phase 5 to 7 and Heart Rate at phase 9 (n=2059).**

| | **Heart rate** | |
|---|---|---|
| **Change† in healthy lifestyle score** | **Model 1 β (95% CI)** | **Model 2 β (95% CI)** |
| Deterioration (n=123) | reference | reference |
| Stable unhealthy (n=484) | -4.28 (-7.28 to -1.29)* | -4.73 (-7.62 to -1.84)* |
| Stable healthy (n=1,349) | -2.07 (-4.20 to 0.07) | -2.165 (-4.72 to -0.59) * |
| Improvement (n=103) | -7.43 (-10.81 to -4.06)** | -8.59 (-11.85 to -5.33)** |
| $p_{overall}$ | ≤0.001 | ≤0.001 |

* $p \leq 0.05$

** $p \leq 0.001$

Model 1: adjusted for age, sex, ethnicity, employment grade and healthy lifestyle score at phase 5

Model 2: adjusted for age, sex, ethnicity, employment grade, healthy lifestyle score at phase 5, presence of any cardiometabolic condition and beta-blockers use at phase 9

†A deterioration and improvement represents a change of at least 2 points in the healthy lifestyle score over 5 years. „Stable unhealthy“ category includes those participants who reported 0 or 1 healthy lifetyle beheaviour at phase 5 and 7. „Stable healthy“ category includes those participants who reported 2, 3 or 4 healthy lifetyle behaviors at phase 5 and 7.
